# Neuronal Biomarkers and Micronutrient Status in Adults with Mild Cognitive Impairment

**DOI:** 10.3390/nu18152428

**Published:** 2026-07-24

**Authors:** Vineela Vepakomma, Basanth Geereddy, Saanvi S. Singireddy, Soumya R. Gurrala, Karthikeyan Ramanujam, G. Bhanuprakash Reddy

**Affiliations:** 1 Department of Biochemistry, ICMR-National Institute of Nutrition, Hyderabad 500007, India; vepakommavineela996@gmail.com (V.V.); saanvi.singireddy@gmail.com (S.S.S.); soumyareddygurrala1701@gmail.com (S.R.G.); 2 Kamineni Academy of Medical Sciences and Research Centre, Hyderabad 500068, India; basanth.geereddy@gmail.com; 3 Department of Clinical Epidemiology, ICMR-National Institute of Nutrition, Hyderabad 500007, India; karthikjpt@gmail.com

**Keywords:** mild cognitive impairment, MoCA, neuronal biomarkers, circulatory micronutrient levels, India

## Abstract

Background: Mild cognitive impairment (MCI) represents an intermediate state between normal cognitive ageing and dementia. Given the increasing dementia burden in the country, there is a critical need for early detection through the assessment of MCI. However, studies that comprehensively examined the associations between micronutrients, neuronal markers, and cognitive impairment are scarce. Methods: A community-based cross-sectional study was conducted among 184 adults aged 55–85 years in Hyderabad between January 2024 and March 2025. Sociodemographic, anthropometric, biochemical, nutritional, and neuronal biomarkers were estimated. Cognitive function was assessed using the Montreal Cognitive Assessment (MoCA) tool. Results: The study reported an MCI prevalence of 36.4% among older adults, significantly associated with higher HbA1c and systolic blood pressure, and elevated neuronal markers such as β-Amyloid, T tau, and Nfl. Vitamin D, B1, B2, B6, and B9 levels were significantly lower in the MCI group, with a higher burden of deficiency. Higher vitamin levels were correlated with better MoCA scores and a lower predicted probability of MCI, especially vitamins D and B6. In particular, B2 and B6 have shown associations with BDNF, tau, and Nfl markers. Conclusions: Lower levels of circulatory vitamins were significantly associated with a higher probability of MCI. Also, neuronal biomarkers were significantly associated with micronutrient deficiencies. These findings suggest that micronutrient deficiencies may be associated with MCI in older adults, highlighting the potential importance of maintaining adequate micronutrient status to support healthy cognitive aging and possibly reduce the risk of dementia. However, given the cross-sectional design of the present study, causal relationships cannot be established. Future longitudinal studies are warranted to validate these associations and further clarify the temporal relationship between micronutrient status and cognitive decline.

## 1. Introduction

India is undergoing accelerated population aging, with a substantial rise in the proportion of individuals aged over 65 years [1]. This demographic transition is accompanied by a rise in the prevalence of chronic non-communicable diseases (NCDs), posing a major threat to the health of the adult and geriatric population. Chronic diseases are estimated to cost the nation up to 4.3 trillion in terms of productivity and health care costs [2,3]. Among the spectrum of existing NCDs, dementia stands out as one of the most prevalent in older adults. Given its size and population diversity, India contributes significantly to this growing epidemic, with estimates of up to 7.5 million cases by 2040 [4,5]. The onset of dementia results from an interplay between genetic factors and environmental influences, including nutrition [6]. While an individual’s genetic predisposition is largely not amenable to modification, several modifiable risk factors, such as physical inactivity, diabetes mellitus, and elevated blood pressure, play important roles in its pathogenesis and can be addressed through changes in diet and lifestyle [7,8].

Mild cognitive impairment (MCI) represents a transitional state between normal cognitive ageing and dementia. It is characterised by impairments in cognitive domains such as language, visual perception, conceptual thinking, memory, and attention [9]. Recently, we reported higher micronutrient deficiencies in individuals with higher risk of dementia [10]. Given the widespread micronutrient deficiencies in India [11,12], and increasing dementia burden in the country, there is a critical need for early disease detection through the assessment of MCI [13]. Although MCI is not diagnostic of dementia, it is regarded as a reliable predictor of its future development, with studies estimating that 10–15% of individuals with MCI eventually progress to dementia [14,15].

Several tools have been developed to detect MCI, among which the Montreal Cognitive Assessment (MoCA) tool is one of the most widely used. MoCA evaluates multiple cognitive domains, including visuospatial ability, executive function, attention, concentration, language, memory, abstraction, and orientation. It demonstrates a high (90%) sensitivity for detecting MCI, attributable to its longer recall period and fewer learning trials compared with other cognitive screening tests [16].

Meanwhile, malnutrition has emerged as a risk factor for cognitive impairment, with inadequacies in several micronutrients contributing to conditions such as hypertension, obesity, and type 2 diabetes mellitus, which are themselves associated with cognitive decline [17]. Micronutrients such as vitamins and minerals are critical for maintaining neuronal health, and imbalances or deficiencies may contribute to neurological disorders [18]. In particular, D and B group vitamins are important in neuronal metabolism, methylation pathways, antioxidant defense, and neurotransmitter synthesis [19,20].

Despite this, very few studies have examined the influence of blood vitamin status on cognitive ageing [7,21]. This represents a critical knowledge gap, particularly in the context of India’s ongoing demographic transition and rising dementia prevalence, underscoring the importance of identifying modifiable factors, particularly nutrient interactions that may preserve cognitive health [22]. Moreover, integration of neuronal biomarkers such as BDNF, β-amyloid, T-tau and neurofilament light chain (Nfl), alongside early cognitive screening, strengthens the biological relevance of the associations between micronutrient status and MCI. Given the limited research in the Indian context using the MoCA to examine the relationship between vitamin profiles and MCI, and particularly the association of vitamin status with neuronal biomarkers, the present study aims to help address this important research gap.

## 2. Materials and Methods

### 2.1. Study Design and Sample Size

From January 2024 to March 2025, we conducted a community-based cross-sectional study among older adults in Hyderabad, Telangana, a southern state in India. The study population comprised men and women aged 55 to 85 years. The sample size was estimated to determine the prevalence of mild cognitive impairment (MCI), assuming a pooled prevalence of 35% from Indian studies [8,23,24,25,26,27]. Using the single-population proportion formula with a 95% confidence level (Z = 1.96), an absolute precision of 10%, a design effect of 1.5, and 30% anticipated non-response and missing data, the final required sample size was 189 participants.

The city was divided into four zones (east, west, north, and south), and, using a simple random sampling procedure, two wards were selected from each zone to capture the city’s population. For the enrolment process, four locations were selected in each ward, and health outreach camps were organised to maximise participant reach, during which participants received a detailed explanation of the study’s purpose. A total of 1020 volunteers were initially assessed for eligibility. Of these, 104 individuals were excluded due to multivitamin supplementation, while 290 members did not meet the inclusion criteria because of chronic diseases, recent infections within the past 3 months, inability to communicate, recent or current hospitalisation, or a history of psychiatric disorders like dementia and depression. Additionally, 342 individuals from the preliminary assessment didn’t volunteer for blood sample collection. Finally, a total of 184 participants were enrolled in the study, comprising 116 males and 68 females. The study was conducted in accordance with the guidelines outlined in the 1964 Declaration of Helsinki and its subsequent amendments.

### 2.2. Data Collection

A self-designed questionnaire documented sociodemographic information from participants, including age, gender, residence, literacy status, current alcohol and smoking consumption, self-reported comorbid conditions, food habits, physical activity, and cohabitation. Food habits were grouped into vegetarian (those who never consumed animal foods, including eggs) and mixed groups, which consumed both animal and plant foods.

### 2.3. Anthropometric Measurements

Body weight and height were recorded, and body mass index (BMI) was calculated as the ratio of weight in kilograms to the square of height in meters. Waist circumference (WC) and hip circumference (HC) were measured using a SECA 201 fiber-reinforced non-elastic tape, and the waist-to-hip ratio (WHR) was calculated. Blood pressure (BP) was measured three times using a digital oscillometer, with 5 min intervals between measurements, and the average of the three readings was taken.

### 2.4. Biochemical Estimations

Following an overnight fast, venous blood samples were drawn at a specified health center or at participants’ homes and transported to the laboratory on ice within 3–4 h. Upon arrival, whole blood samples were aliquoted, and the remaining samples were centrifuged at 3500 rpm for 15 min at 4 °C to separate plasma within 4 h. All samples were stored at −80 °C for further investigation.

From the whole blood sample, fasting blood glucose (FBG) and glycated haemoglobin (HbA1c) were measured using a glucometer (Accu-Chek^®^ Active Roche Diagnostics GmbH, Mannheim, Germany) and an Affinion AS100 Analyser (Abbott, Chicago, IL, USA), respectively. Total haemoglobin was quantified by the cyanmethemoglobin method using a Shimadzu UV-2600 (Kyoto, Japan) spectrophotometer. Using enzymatic methods, plasma lipid profiles were assessed with the LDX Analyzer (Abbott, Chicago, IL, USA). Renal function was evaluated by measuring plasma creatinine levels using the modified kinetic Jaffe method with a Biosystems kit (Barcelona, Spain).

### 2.5. Circulatory Nutritional and Neuronal Markers

Plasma levels of vitamins A and D were determined using reverse-phase HPLC coupled with ultraviolet detection [28,29]. The active coenzyme forms of vitamins B1 (thiamine pyrophosphate) and B2 (flavin adenine dinucleotide) were measured in whole blood, while the active form of vitamin B6 (pyridoxal-5′-phosphate) was quantified in plasma by utilizing commercially available HPLC kits with fluorescence detection (Recipe Chemicals+ Instruments GmbH, Munich, Germany) [12]. Blood samples underwent protein precipitation, stabilisation and post-column derivatization (wherever applicable) prior to chromatographic separation. Quantification was carried out by comparing analyte retention times and peak areas against certified standards according to the manufacturer’s protocol. Plasma folate and B12 levels were estimated using an immunoanalyzer (Beckman Coulter Unicel DXI 800, version 7.1.0, Brea, CA, USA) using a competitive binding chemiluminescence method. Plasma beta-amyloid (1–42) (sensitivity: 9.38 pg/mL, detection range: 15.63–1000 pg/mL), Nfl (sensitivity: 9.38 pg/mL, detection range: 15.63–1000 pg/mL), and microtubule-associated total Tau levels (T-tau) (sensitivity: 4.69 pg/mL, detection range:7.81–500 pg/mL) were measured using Novus Biologicals (Minneapolis, MN, USA) Enzyme-linked immunosorbent assay (ELISA) kits: NBP2-69913, NBP2-81184, and NBP2-62749, respectively. These kits are based on the sandwich ELISA principle, in which the micro-ELISA plate was pre-coated with an antibody specific to humans. Standards and samples were added, followed by a biotinylated detection antibody. Plasma brain-derived neurotrophic factor (BDNF) levels were measured using R&D Systems ELISA Duoset (DY248) that utilizes a solid-phase sandwich ELISA method with a detection range of 23.4–1500 pg/mL.

### 2.6. Montreal Cognitive Assessment (MoCA)

The MoCA is one of the most commonly used cognitive screening tests worldwide for detecting MCI [30]. It evaluates many cognitive domains and takes 10 min to administer. The MoCA is available in more than 60 languages and dialects, including Indian languages. This tool was adapted and validated in many Indian languages, including Telugu, and the diagnostic accuracy of the Indian MoCA version was reported to have excellent sensitivity and specificity in detecting MCI [31]. This tool assesses 8 cognitive domains, such as visuospatial/executive function (clock drawing, cube copying, alternate trial making task), naming (three-item confrontation naming task), language (phonemic fluency, verbal abstraction task), memory, delayed recall, attention (target detection by tapping, serial subtraction), abstraction (similarity between items), and orientation (time and place). The maximum score is 30 points, with an additional point added to the final score for those with <12 years of education. The primary author had finished the mandatory training conducted by the MoCA team to administer and score the Telugu version of the MoCA test.

Although the commonly used cut-off for MoCA-based MCI screening is 26, some studies have widely recommended that this cut-off leads to inflated false-positive rates and have reported that a cut-off of 23 yields the best diagnostic accuracy [32]. A recently published systematic review evaluating the accuracy of MoCA for detecting MCI also supported a lower cut-off [33]. Moreover, a study from the Indian population recommended that with a cut-off of 26, the sensitivity was high, but the specificity was quite low, suggesting a higher referral burden for detailed neuropsychological testing, and reported that a lower cut-off of 23 yields optimal sensitivity and specificity, with good diagnostic accuracy (70.8%) [16]. Therefore, a cut-off of <23 is used in the present study to screen for MCI using the MoCA tool and to further associate it with nutritional and neuronal markers.

### 2.7. Statistical Analysis

Data processing and statistical analyses were performed using R version 4.3.2 (R Foundation for Statistical Computing, Vienna, Austria) and GraphPad Prism 8. Prior to analysis, continuous concentrations of vitamin biomarker were screened for potential outliers using graphical methods and percentile distributions. Observations at or around the upper 1% of the distribution were reviewed for biological plausibility, and only confirmed extreme values were excluded. The normality of continuous variables, including skewness and kurtosis, was assessed using the Shapiro–Wilk test. Normally distributed variables were presented as mean ± standard deviation (SD), whereas biochemical and blood vitamin levels were expressed as median and interquartile range (IQR). Group comparisons between participants with MCI and without MCI were performed using Student’s *t*-test and the Mann–Whitney U test based on data distribution. The Chi-square test was used to examine associations between categorical variables and MCI status. To assess the cumulative burden of vitamin deficiencies, a deficiency score was assigned to each participant by summing the number of vitamin deficiencies (vitamin D, vitamin B1, vitamin B2, vitamin B6, vitamin B9, and vitamin B12 (range: 0–6)) which resulted in a median deficiency score of 2 in the study population. Hence, participants were categorized into <2 and ≥2 deficiency groups for subsequent analyses. Spearman’s rank correlation coefficient was calculated to assess the association between circulating vitamin levels and MoCA scores, treated as continuous variables. Multivariable logistic regression analyses were performed to evaluate the association between individual vitamin levels and MCI status, adjusting for potential confounders, including conventional demographic and clinical risk factors. Further, adjusted logistic regression models were used to estimate the predicted probabilities of MCI across different vitamin levels, and the results were visualized with 95% confidence intervals and rug plots to display the distribution of observations.

To evaluate the discriminatory ability of vitamins in predicting MCI, receiver operating characteristic (ROC) curve analysis was performed, and the area under the curve (AUC) with 95% confidence intervals (CI) was calculated for individual vitamins and combined vitamin models. Statistical significance was considered at a two-sided *p*-value < 0.05.

## 3. Results

**Figure 1:**
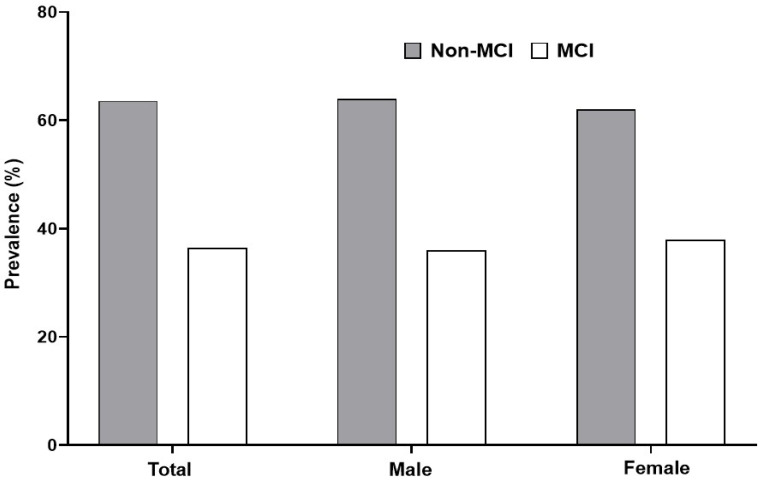
Prevalence of MCI in the total study population and stratified by gender.

**Figure 2:**
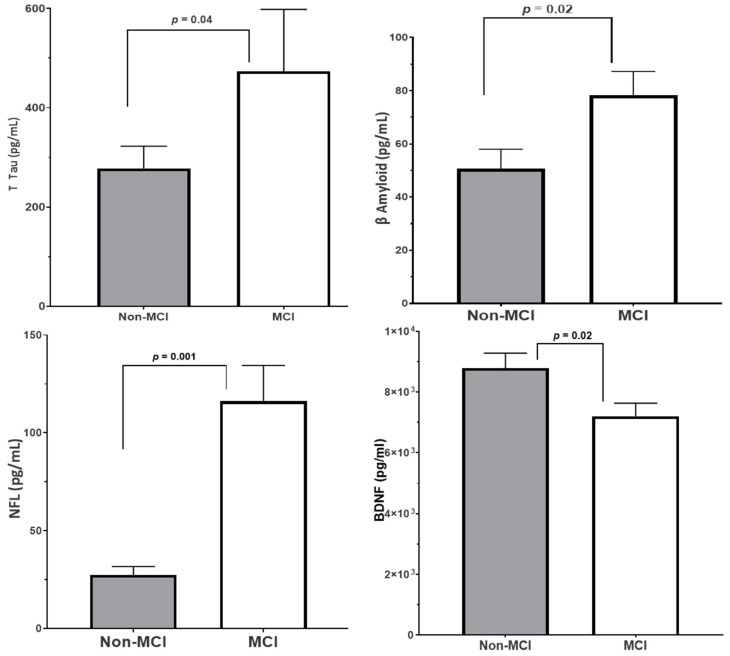
Comparison of neuronal biomarkers between non-MCI and MCI groups. *p* < 0.05 is considered statistically significant.

The socio-demographic and clinical characteristics of the study groups are shown in Table 1. Among the study participants, men and women account for 63% and 37%, respectively. MoCA scores showed a declining trend with increasing age. Based on the MoCA tool, the prevalence of MCI in the study population was found to be 36.4%, which did not differ much by gender (Figure 1). Significantly higher levels of neurodegenerative markers, along with lower BDNF concentrations, were observed in the MCI group, complementing the validity of the MoCA-based classification in identifying individuals with early cognitive impairment (Figure 2). The study population is predominantly literate (95%) and has more than 12 years of education. Mixed diet consumption was higher in the MCI group (69%) compared to the non-MCI group (56%). The majority of people lived with family, with a 4.5% proportion of the MCI group living alone compared to the non-MCI group, which is 1.7%. No differences were observed between the MCI and non-MCI groups with respect to alcohol intake and smoking patterns.

The median age of the study population is 65 years, with 67 years in the MCI group and 63 years in the non-MCI group (*p* = 0.01). HbA1c (*p* = 0.0008) and systolic blood pressure (*p* = 0.01) were significantly higher in the MCI group compared to the non-MCI group. Other variables did not differ much between the groups (Table 1).

Neuronal markers such as β-amyloid (*p* = 0.02), T-tau (0.04), and Nfl (*p* = 0.001) were significantly higher in the MCI group compared to the non-MCI group. Neuroprotective marker, BDNF, was significantly lower in the MCI group compared to the non-MCI group (*p* = 0.02) (Figure 2).

### 3.1. Comparison of the Vitamin Levels Between Groups

**Figure 3:**
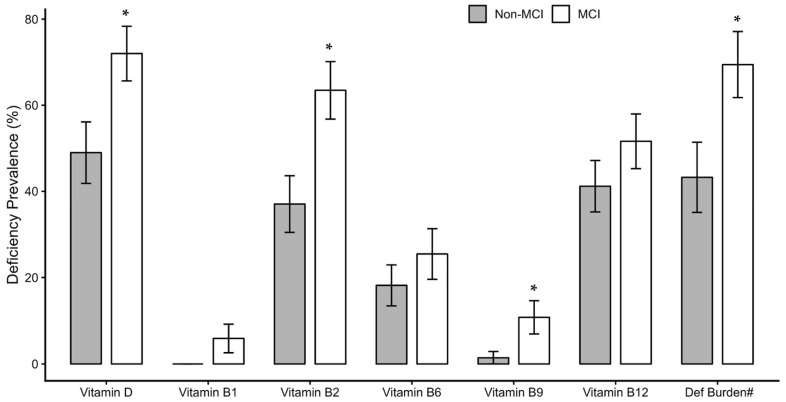
Prevalence of vitamin deficiencies in the non-MCI and MCI groups, expressed as % with SEM. The chi-square test was used to compare vitamin deficiencies between the groups. * Indicates the statistical significance between the groups (*p* < 0.05). # indicates the cumulative burden of vitamin deficiency based on the median deficiency score.

Plasma and whole-blood vitamin levels were estimated and presented in Table 2. Vitamins D, B1, B2, B6, B9, and B12 levels were significantly lower in the MCI group compared to the non-MCI group. The prevalence of vitamin deficiencies was estimated. Deficiencies of vitamin D (72% vs. 48%), B2 (63% vs. 37%), and B9 (11% vs. 2%) were significantly higher in the MCI group compared to the non-MCI group. Deficiencies of B1 (6% vs. 2%), B6 (25% vs. 18%), and B12 (52% vs. 41%) vitamins were higher in the MCI group than in the non-MCI group, but not significantly different. Further, the cumulative burden of vitamin deficiency was calculated for 6 vitamins. With a median cut-off of 2, we found that the frequency of deficiencies (>2) was higher in the MCI group (39%) than in the non-MCI group (19%). Figure 3 demonstrates the prevalence of vitamin deficiencies between the MCI and non-MCI groups.

### 3.2. Associations Between Circulatory Vitamins and MCI

**Figure 4:**
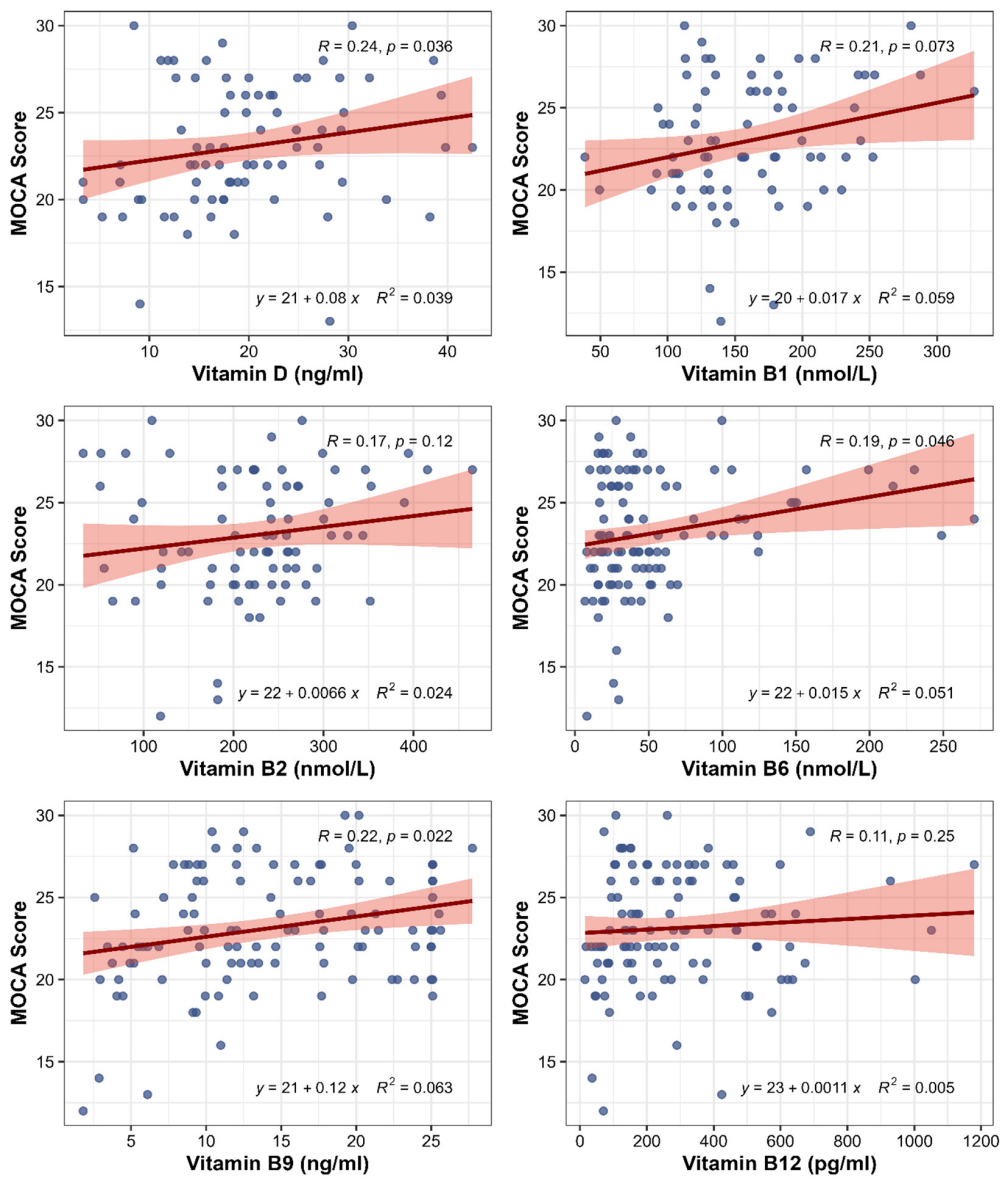
Scatter plots showing the association of vitamin levels with MoCA scores. Blue dots represent the individual values.

Figure 4 shows the correlation coefficients between MoCA scores and blood vitamin levels. At higher levels of vitamin D (r = 0.24, *p* = 0.04), B6 (r = 0.2, *p* = 0.04), and B9 (r = 0.22, *p* = 0.02), we observed significantly higher MoCA scores. Similarly, weak relationships with MoCA score were identified for vitamin B1 (r = 0.21, *p* = 0.07), B2 (r = 0.17, *p* = 0.12), and B12 (r = 0.11, *p* = 0.25).

### 3.3. Adjusted Relationships Between Blood Vitamin Levels and MCI Probability

**Figure 5:**
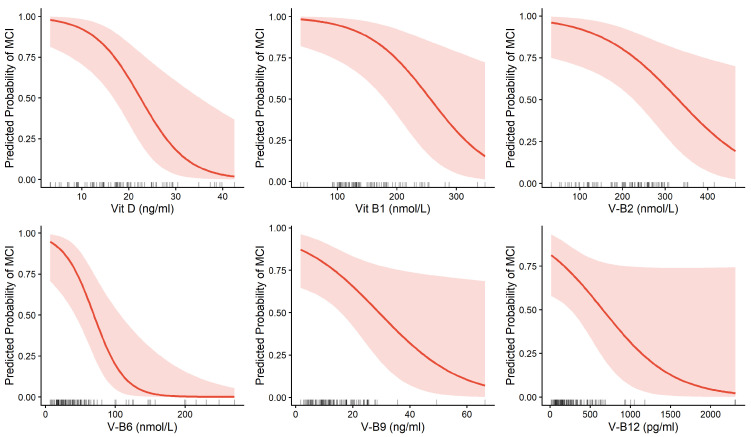
Adjusted relationships between vitamin levels and predicted probability of MCI. All the models adjusted for Age, Sex, Years of education, BMI, HbA1C, SBP, T tau, and Nfl.

Figure 5 presents the adjusted dose-response relationships between circulating vitamin levels and the predicted probability of MCI derived from multivariable logistic regression models adjusted for age, sex, education, BMI, HbA1c, SBP, T-tau, and Nfl levels. Higher vitamin concentrations were associated with lower predicted probability of MCI across all nutrients. For vitamin D, the probability decreased from 0.94 (95% CI: 0.74–0.99) at the 10th percentile (8.3 ng/mL) to 0.20 (95% CI: 0.04–0.61) at the 90th percentile (29.5 ng/mL). Vitamin B6 showed a similar pattern, with the probability decreasing from 0.92 (95% CI: 0.66–0.99) at lower concentrations (15.8 nmol/L) to 0.04 (95% CI: 0.004–0.36) at higher concentrations (136.4 nmol/L), suggesting a marked non-linear association.

Vitamin B1 and vitamin B2 were also inversely associated with MCI probability, although the reduction was more gradual. For vitamin B1, the predicted probability decreased from 0.95 (95% CI: 0.73–0.99) to 0.57 (95% CI: 0.21–0.87) across the observed range. Similarly, for vitamin B2, the probability declined from 0.93 (95% CI: 0.70–0.99) to 0.57 (95% CI: 0.28–0.83). Folate (vitamin B9) showed a modest decrease from 0.84 (95% CI: 0.62–0.95) at lower levels (5.2 ng/mL) to 0.57 (95% CI: 0.31–0.80) at higher levels (25.1 ng/mL), indicating a relatively linear association. Vitamin B12 demonstrated a similar trend, with predicted probabilities decreasing from 0.79 (95% CI: 0.57–0.92) at low concentrations (69.1 pg/mL) to 0.51 (95% CI: 0.24–0.78) at higher concentrations (636.2 pg/mL). Overall, the inverse relationship between vitamin levels and MCI probability was consistent across all nutrients, with steeper reductions observed for vitamin D and vitamin B6.

### 3.4. Receiver Operating Curve (ROC) Analysis

Table A1 shows the results of receiver operating characteristic analysis used to assess how well individual vitamins and their combinations distinguish between individuals with and without MCI. Among the individual vitamins, ROC curves showed that vitamin D alone had the highest area under the curve (AUC) at 0.67 (95% CI: 0.57 to 0.78). Among models with two vitamins, the combination of vitamins B6 and D yielded an AUC of 0.73 (95% CI: 0.62 to 0.83), and the inclusion of three (B6, B12, and D) showed a much higher AUC of 0.76 (95% CI: 0.66 to 0.86). The combination of all 5 vitamins yielded the highest AUC of 0.78 (95% CI: 0.68 to 0.88). Overall, individual vitamins showed limited ability to distinguish MCI, but combining them, especially vitamins D, B2, and B6, consistently improved model performance. Table A1 provides AUC values for additional individual micronutrients and combinations.

### 3.5. Neuronal Biomarkers vs. Vitamins

**Figure 6:**
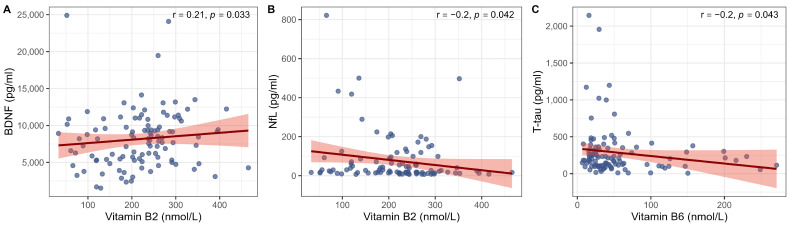
Scatter plots showing the association of B vitamins with neuronal markers. (**A**) Scatter plot showing the correlation of vitamin B2 with BDNF, (**B**) Scatter plot showing the correlation of vitamin B2 with NfL (**C**) Scatter plot showing the correlation of vitamin B6 with T-tau. Blue dots represent the individual values.

Correlation coefficients were calculated to explore associations between neuronal and nutritional markers (Figure 6). At higher B2 levels, there is a significant increase in the neuroprotective marker BDNF (r = 0.2, *p* = 0.03). A significant inverse association was shown between vitamin B2 and Nfl markers (r = −0.2, *p* = 0.04). Similarly, as vitamin B6 levels increase, T-tau plasma concentrations significantly decrease (r = −0.2, *p* = 0.04). No significant associations were found between other nutrients and neuronal markers.

## 4. Discussion

In the present study, 37% of participants were identified as having MCI on administration of MoCA. Lifestyle disorders such as diabetes and hypertension were significantly higher in the MCI group compared to the non-MCI group. Importantly, this study revealed a significant association between circulatory vitamin levels (vitamin D, B2, B6, B9, and B12) and MCI. This finding was consistent with a recent study that reported micronutrient levels were significantly associated with the risk of MCI [34]. However, the reported findings were based on dried blood samples, whereas the current study findings are based on venous blood samples, which are likely to be more accurate and reliable. Although earlier studies have reported associations between individual nutrients and cognitive dysfunction, measured using various tools [35,36,37,38], no studies have linked circulatory vitamin status to MoCA and neuronal markers. Additionally, we reported the prevalence of vitamin deficiencies in both MCI and non-MCI groups and found that deficiencies in vitamin D, B2, and B9 were significantly higher in the MCI group than in the non-MCI group. Furthermore, the cumulative burden of vitamin deficiencies was significantly higher in the MCI group than in the non-MCI group.

In this study, higher levels of vitamin D and B-group vitamins were associated with a lower predicted probability of MCI as assessed by MoCA, even after adjusting for potential confounders. However, the wider confidence intervals at higher concentrations reflect the smaller number of observations in these ranges. Micronutrients are reported to play an important role in cognitive ageing—for instance, vitamin A, through its active metabolite retinoic acid, is involved in synaptic plasticity and amyloid processing, and also impaired retinoid signalling has been implicated in Alzheimer’s disease progression [39]. Riboflavin supports mitochondrial energy metabolism and antioxidant pathways, which are crucial for preventing neurodegeneration [40]. Vitamin B6 deficiency has been associated with altered neurotransmitter metabolism and dysregulated noradrenergic signalling, leading to behavioural deficits and cognitive dysfunction [41]. Folate, along with vitamin B12, is essential for one-carbon metabolism, DNA synthesis, methylation reactions, and homocysteine regulation. They also play a key role in regulating methyl group availability in the brain, thereby influencing neuronal integrity and cognitive performance [42]. Additionally, deficiency of vitamin B12 has been linked with cognitive decline, impaired memory, and increased risk of neurodegeneration in older adults. Previous studies have reported associations between lower vitamin B12 concentrations and poor cognitive functioning, particularly in memory and executive function [43].

Further, the observed associations of BDNF and Nfl with vitamin B2, and of T-tau with vitamin B6, suggest that micronutrient status may influence early neurodegenerative processes underlying cognitive impairment. Vitamin B2 is involved in antioxidant defence and mitochondrial energy metabolism and may modulate BDNF-related neuroprotective pathways in animal models [44]. Although direct evidence linking B2 and T-tau is limited, impaired riboflavin status may contribute to oxidative stress and tau-related neuronal injury [45]. Similarly, the association between B6 and Nfl may reflect the influence of B-vitamin inadequacy on neuronal integrity, as Nfl is a well-established marker of axonal injury and early cognitive decline [46]. The concordance between MoCA-based classification and observed alterations in established neuronal biomarkers supports the utility of combining cognitive assessment with biomarker profiling for identifying early MCI. Given the limited existing literature, these findings may be interpreted as biologically plausible, exploratory associations warranting confirmation in longitudinal studies.

Collectively, these findings corroborate the current study and suggest that inadequate micronutrient status may be associated with early cognitive decline through multiple interconnected pathways. Additionally, the B2-deficient group has significantly lower MoCA scores in naming (*p* = 0.03), attention (*p* = 0.009), and language domains (*p* = 0.007) compared to the normal group. Also, B9-deficient individuals’ performance in the attention (*p* = 0.01), delayed recall (*p* = 0.02), and orientation domains (*p* = 0.02) was significantly poorer than that of normal individuals (Table A2).

ROC analysis revealed that models with a combination of four vitamins performed better, with vitamin B2, B6, B12, and D, with the highest AUC of 0.78, but adding B9 did not lead to further improvement, as the model including all vitamins had an AUC of 0.78. Nevertheless, it is important to consider the potential synergistic interactions among micronutrients, as nutrients with modest individual effects may collectively contribute to the overall association with cognitive impairment.

Limitations: As a cross-sectional study, it limits the ability to establish temporality and causal relationships and may introduce selection bias. Further, the study was not designed or adequately powered to perform detailed gender-based analyses. Thus, studies involving larger longitudinal cohorts are required to validate these findings and to evaluate the combined effects of micronutrient profiles on predictive models. Additionally, inclusion of other nutritional factors, such as minerals and broader neurodegenerative biomarkers, may provide a more in-depth understanding of the nutritional determinants of cognitive decline.

## 5. Conclusions

In summary, this study examines the association between the circulatory micronutrient profile and MCI, as classified using the MoCA, while also exploring its relationship with neuronal markers. Evidence linking micronutrients to neuronal markers remains limited, and the present findings help address this important research gap. In this study, 37% of participants were classified as MCI, and lower vitamin concentrations were associated with a higher predicted probability of MCI. The observed associations highlight the potential importance of adequate micronutrient status in early cognitive ageing. Given the limited effectiveness of currently available treatments for dementia, identification and prevention of cognitive decline during the MCI stage could offer a more effective strategy for reducing the disease burden. These findings may have important implications for public health approaches aimed at promoting healthy brain aging through early nutritional interventions and risk-reduction strategies.

## Figures and Tables

**Table 1:** Comparison of socio-demographic, anthropometric, and biochemical profiles between non-MCI and MCI groups.

Variables	Total (*N* = 184)	Non-MCI (*n* = 117)	MCI (*n* = 67)	*p*-Value
Age (years)	64.5 (61–70)	63 (59–69.5)	67 (62–73)	0.01
Education (years)	15 (14–17)	15 (14–17)	14 (12–17)	0.02
Current alcohol Consumption Yes No	51 (27.7%) 133 (72.3%)	32 (27.3%) 85 (72.6%)	19 (28.3%) 48 (71.6%)	0.88
Current smoking status Yes No	4 (1.6%) 180 (97.8%)	3 (2.5%) 114 (97.4%)	1 (0.6%) 66 (98.5%)	0.63
Food habits Vegetarian Mixed diet	74 (40.2%) 110 (59.8%)	53 (45.3%) 64 (55.7%)	21 (31.3%) 46 (68.6%)	0.17
Living status With Family Alone	179 (97.3%) 5 (2.7%)	115 (98.3%) 2 (1.7%)	64 (95.5%) 3 (4.5%)	0.26
	Median (IQR)	Median (IQR)	Median (IQR)	*p*-value
Height (m)	1.64 (1.57–1.69)	1.65 (1.57–1.70)	1.61 (1.55–1.67)	0.05
Weight (kg)	70 (62.7–78.5)	68.8 (63–77.4)	72.5 (61.1–79)	0.53
Body mass index (kg/m^2^)	26.2 (23.5–29.6)	25.6 (23.2–29.4)	26.7 (24.2–30.8)	0.09
Waist circumference (cm)	99.5 (92.0–104)	99.0 (92.0–104)	100 (91.0–105)	0.62
Hip circumference (cm)	106 (99.0–114)	107 (99.0–113)	105 (98.0–116)	0.78
Waist–hip ratio (WHR)	0.93 (0.88–0.96)	0.91 (0.87–0.95)	0.93 (0.89–0.97)	0.18
Fasting blood glucose (mg/dL)	114 (99–139)	111 (98.0–127)	118 (101–146)	0.08
HbA1c (%)	6.3 (5.8–7.5)	6.1 (5.7–6.8)	6.9 (6.1–8.3)	0.0008
Haemoglobin (g/dL)	12.7 (11.4–13.7)	12.7 (11.2–13.6)	12.6 (11.6–14.2)	0.48
Systolic blood pressure (mm Hg)	136 (124–148)	131 (124–144)	139 (125–155)	0.01
Diastolic blood pressure (mm Hg)	81.5 (74.7–88.0)	81.5 (74.5–87.7)	81.5 (74.7–88.0)	0.87
Plasma creatinine (mg/dL)	0.9 (0.8–1.1)	1.0 (0.78–1.17)	0.9 (0.8–1.0)	0.45
Total cholesterol (mg/dL)	162 (130–193)	154 (127–186)	168 (137–195)	0.18
Triglycerides (mg/dL)	108 (84.0–140)	105 (84.0–129)	114 (83.1–147)	0.49
Low-density lipoprotein (mg/dL)	101 (78.0–135)	96.5 (71.0–128)	113 (82.0–136)	0.16
High-density lipoprotein (mg/dL)	36.0 (28.5–42.0)	32.5 (27.0–43.0)	37.0 (31.0–42.0)	0.16

Values are represented as the median with the interquartile range. Variable comparisons between groups were performed using the Mann–Whitney U Test. Categorical variables, including gender, education, alcohol intake, smoking status, food habits, and living status, were presented as frequencies (%), and comparisons were performed using a Chi-square test; *p* < 0.05 was considered statistically significant.

**Table 2:** Median (25th–75th percentile) blood levels of vitamins in the study population.

Vitamins	Total (*n* = 184) (Median, IQR)	Non-MCI (*n* = 117) (Median, IQR)	MCI (*n* = 67) (Median, IQR)	*p*-Value
Vitamin A µmoles/L	3.2 (2.5–4.2)	3.1 (2.27–3.8)	3.4 (2.6–4.65)	0.10
Vitamin D ng/mL	17.7 (12.6–25.2)	20.9 (15.2–28.1)	16.2 (9.2–20.6)	0.001
Vitamin B1 nmol/L	139 (118–182)	159 (121–197)	136 (108–178)	0.05
Vitamin B2 nmol/L	226 (167–271)	243 (187–301)	203 (139–250)	0.006
Vitamin B6 ^#^ nmol/L	36.3 (22.4–60.0)	38.6 (24.3–107)	29.6 (18.9–49.7)	0.006
Vitamin B9 ^#^ ng/mL	13.5 (9.1–21.4)	16.5 (9.9–25.0)	11.4 (6.1–19.8)	0.002
Vitamin B12 ^#^ pg/mL	227 (125–465)	265 (142–463)	179 (80.2–498)	0.06

Values are represented as median with interquartile range (IQR). The association of vitamin levels between the groups was tested using a *t*-test for parametric data, and a Mann–Whitney U test for non-parametric data, denoted by #. *p* < 0.05 was considered statistically significant.

## Data Availability

The data that support the findings of this study are available from the corresponding author, GBR, upon reasonable request.

## Abbreviations

The following abbreviations are used in this manuscript:

MCI	Mild cognitive impairment
MoCA	Montreal Cognitive Assessment
NCDs	Non-communicable diseases
BMI	Body mass index
WC	Waist circumference
HC	Hip circumference
WHR	Waist-to-hip ratio
BP	Blood pressure
HbA1c	Glycated hemoglobin
FBG	Fasting blood glucose
NFL	Neurofilament light chain
BDNF	Brain-derived neurotrophic factor
SD	Standard deviation
IQR	Interquartile range
AUC	Area under the curve
ROC	Receiver operating characteristic

## Appendix A

**Table A1:** Discriminatory performance of individual vitamins and their combinations for identifying MCI–ROC analysis.

Model	AUC	95% CI	
V-B2	0.66	0.55	0.76
V-B6	0.64	0.54	0.74
V-B9	0.65	0.55	0.74
V-B12	0.59	0.49	0.69
V-D	0.67	0.57	0.78
V-B2 + V-B6	0.70	0.60	0.81
V-B2 + V-B9	0.69	0.59	0.79
V-B2 + V-B12	0.65	0.54	0.76
V-B2 + V-D	0.72	0.62	0.82
V-B6 + V-B9	0.68	0.58	0.77
V-B6 + V-B12	0.66	0.56	0.76
V-B6 + V-D	0.72	0.62	0.83
V-B9 + V-B12	0.64	0.55	0.74
V-B9 + V-D	0.68	0.57	0.78
V-B12 + V-D	0.68	0.57	0.79
V-B2 + V-B6 + V-B9	0.72	0.62	0.83
V-B2 + V-B6 + V-B12	0.71	0.60	0.82
V-B2 + V-B6 + V-D	0.75	0.64	0.85
V-B2 + V-B9 + V-B12	0.67	0.56	0.78
V-B2 + V-B9 + Vit D	0.71	0.60	0.81
V-B2 + V-B12 + Vit D	0.72	0.61	0.82
V-B6 + V-B9 + V-B12	0.68	0.58	0.77
V-B6 + V-B9 + Vit D	0.72	0.61	0.82
V-B6 + V-B12 + Vit D	0.76	0.66	0.86
V-B9 + V-B12 + Vit D	0.68	0.57	0.79
V-B2 + V-B6 + V-B9 + V-B12	0.71	0.61	0.82
V-B2 + V-B6 + V-B9 + Vit D	0.75	0.65	0.85
V-B2 + V-B6 + V-B12 + Vit D	0.78	0.68	0.88
V-B2 + V-B9 + V-B12 + Vit D	0.72	0.61	0.82
V-B6 + V-B9 + V-B12 + Vit D	0.76	0.66	0.86
V-B2 + V-B6 + V-B9 + V-B12 + Vit D	0.78	0.69	0.88

**Table A2:** Comparison of cognitive domains between the vitamin deficiency and normal groups.

**Vitamins**	**Visuospatial**	**Naming**	**Attention**	**Language**	**Abstraction**	**Delayed Recall**	**Orientation**
Vitamin D							
Normal	3.38 (0.96)	2.46(0.55)	5.07 (1.17)	2.1 (0.88)	1.46 (0.68)	3.02 (1.44)	5.53 (0.94)
Deficient	2.96 (1.26)	2.56 (0.56)	4.86 (1.04)	1.91 (0.92)	1.50 (0.65)	2.58 (1.56)	5.76 (0.56)
*p* value	0.08	0.36	0.35	0.32	0.77	0.15	0.13
Vitamin B1							
Normal	3.14 (1.17)	2.5 (0.56)	5.0 (1.11)	2.01 (0.87)	1.46 (0.67)	2.75 (1.58)	5.62 (0.83)
Deficient	3.0 (1.0)	2.3 (0.57)	4.66 (0.57)	1.0 (1.0)	1.33 (0.57)	2.66 (2.3)	6.0 (0.0)
*p* value	0.83	0.614	0.608	0.05	0.73	0.93	0.44
Vitamin B2							
Normal	3.2 (1.16)	2.64 (0.52)	5.24 (0.85)	2.28 (0.66)	1.58 (0.53)	2.6 (1.58)	5.69 (0.72)
Deficient	3.11 (1.71)	2.41 (0.56)	4.69 (1.24)	1.71 (0.96)	1.41 (0.74)	2.84 (1.56)	5.56 (0.88)
*p* value	0.67	0.03	0.009	0.0007	0.18	0.42	0.40
Vitamin B6							
Normal	3.15 (1.14)	2.63 (0.5)	4.97 (1.07)	2.03 (0.83)	1.53 (0.63)	2.88 (1.51)	5.74 (0.56)
Deficient	3.3 (1.08)	2.57 (0.5)	4.96 (1.14)	2.07 (0.89)	1.5 (0.7)	2.65 (1.74)	5.53 (1.02)
*p* value	0.55	0.626	0.94	0.8	0.79	0.5	0.17
Vitamin B9							
Normal	3.11 (1.19)	2.56 (0.55)	4.98 (1.06)	2.06 (0.84)	1.48 (0.67)	2.8 (1.59)	5.73 (0.67)
Deficient	2.62 (0.91)	2.37 (0.51)	4.0 (1.51)	1.5 (1.19)	1.62 (0.74)	1.5 (1.19)	5.12 (1.35)
*p* value	0.25	0.34	0.01	0.07	0.58	0.025	0.02
Vitamin B12							
Normal	3.11 (1.13)	2.6 (0.52)	4.85 (1.06)	2.05 (0.83)	1.54 (0.65)	2.81 (1.6)	5.7 (0.68)
Deficient	3.06 (1.24)	2.5 (0.59)	5.01 (1.14)	2.03 (0.93)	1.41 (0.72)	2.61 (1.62)	5.7 (0.78)
*p* value	0.82	0.31	0.412	0.87	0.29	0.48	0.99

Values are represented as mean with SD. The association of vitamin levels with MoCA cognitive domains was tested using a *t*-test, and *p* < 0.05 was considered statistically significant.
